# Influence of Dietary Experience on the Induction of Preference of Adult Moths and Larvae for a New Olfactory Cue

**DOI:** 10.1371/journal.pone.0136169

**Published:** 2015-08-19

**Authors:** Christophe Petit, Bruno Le Ru, Stéphane Dupas, Brigitte Frérot, Peter Ahuya, Laure Kaiser-Arnauld, Myriam Harry, Paul-André Calatayud

**Affiliations:** 1 Noctuid Stem Borer Biodiversity team, Institut de Recherche pour le Développement c/o icipe, African Insect Science for Food and Health, Nairobi, Kenya; 2 Unité Mixte de Recherche 247, Evolution, Génomes, Comportement et Ecologie, Institut de Recherche pour le Développement, Centre National de la Recherche Scientifique, Gif sur Yvette Cedex, France; 3 Université Paris-Sud, Orsay, France; 4 Unité Mixte de Recherche 1392, Institut d’Ecologie et des Sciences de l’Environnement de Paris, Institut National de la Recherche Agronomique, Versailles, France; Swedish University of Agricultural Sciences, SWEDEN

## Abstract

In Lepidoptera, host plant selection is first conditioned by oviposition site preference of adult females followed by feeding site preference of larvae. Dietary experience to plant volatile cues can induce larval and adult host plant preference. We investigated how the parent’s and self-experience induce host preference in adult females and larvae of three lepidopteran stem borer species with different host plant ranges, namely the polyphagous *Sesamia nonagrioides*, the oligophagous *Busseola fusca* and the monophagous *Busseola nairobica*, and whether this induction can be linked to a neurophysiological phenotypic plasticity. The three species were conditioned to artificial diet enriched with vanillin from the neonate larvae to the adult stage during two generations. Thereafter, two-choice tests on both larvae and adults using a Y-tube olfactometer and electrophysiological (electroantennography [EAG] recordings) experiments on adults were carried out. In the polyphagous species, the induction of preference for a new olfactory cue (vanillin) by females and 3^rd^ instar larvae was determined by parents’ and self-experiences, without any modification of the sensitivity of the females antennae. No preference induction was found in the oligophagous and monophagous species. Our results suggest that lepidopteran stem borers may acquire preferences for new olfactory cues from the larval to the adult stage as described by Hopkins’ host selection principle (HHSP), neo-Hopkins’ principle, and the concept of ‘chemical legacy.’

## Introduction

In Lepidoptera, host plant selection is a crucial event for progeny survival and fitness [1]. Host plant selection is usually determined by the ovipositing female [1,2]. However many species of Lepidoptera have highly mobile larvae that can engage in host plant selection [3]. The larvae possess olfactory receptors which play an important role in discriminating odours that emanate from different plants, thus allowing for host-plant recognition and selection [4]. In this context, host plant volatile compounds are essential for distant attraction to the host plant and are involved in both oviposition and feeding sites preferences.

Experience based upon sensory sampling of the environment allows the insects to adapt to new conditions [5], as a result of a behavioural [5] and neurophysiological [6] phenotypic plasticity. It is widely accepted that dietary experience can influence the host plant selection in insects. The theory of larval memory, previously named as Hopkins’ host selection principle (HHSP) [7], postulates that (i) adult insects demonstrate a preference for the host plant species on which they developed as larvae and (ii) a memory of the feeding substrate is stored in the central nervous system and transferred through metamorphosis to the adult stage. However, there is still scarce evidence (e.g. [8–10]) for pre-imaginal conditioning of host choice as suggested by HHSP since metamorphosis involves a major restructuration of the central nervous system, which should "erase" the chemical memory stored by the larvae (see [11] for review). Alternatively, the concept of chemical legacy by Corbet [12] presumes that the chemical fingerprint of the host plant can be stored in the hemolymph of the larva and then at the surface of the pupae. Thus, when the young adults emerge, they detect the chemical signals issued from the host plant on the surface of the pupae, which cause changes in the subsequent behaviour of the adult. Experience during the imago stage can also induce a preference; this concept is named neo-Hopkins principle [13]. Imaginal experience has been demonstrated to induce oviposition site preferences in some Lepidoptera species [14–16]. However, both pre-imaginal and imaginal experiences on subsequent oviposition and feeding behaviours continue to be controversial and HHSP and chemical legacy have yet to be clearly demonstrated or negated [17].

In this study, we explore these concepts by investigating the effects of dietary experience with vanillin on the subsequent adult and larval preference for vanillin, taking into consideration the host plant range of the species. The effects of dietary experience may differ according to the host range given that poly-, oligo- and monophagous species use different host selection strategies. For example, Bernays [18] argues that oligophagous and monophagous species are more efficient when making decision for host selection than polyphagous species. We also investigated whether transfer of information from adults to offspring took place. Behavioural (Y-tube olfactometer assays) and electrophysiological (electroantennography [EAG]) experiments were carried out using a mono-, oligo- and polyphagous noctuid stem borer species.

## Material and Methods

### Insects

Three lepidopteran stem borer species belonging to the Noctuidae, which differ in their host plant range, were used in this study.

*Busseola nairobica* Le Ru recently described by Félix et al. [19] is a monophagous species feeding on the broadleaf panicum (*Panicum deustum* Thunb 1794) [20–23]. The *B*. *nairobica* larvae used were collected from *P*. *deustum* in the Ngong forest (Nairobi, Kenya). The developmental time from egg to adult emergence lasts about 90 days at 26 ± 1°C (Petit, C., Pers. Obs.).

*Busseola fusca* (Fuller 1901) is an oligophagous species found on nine plant species belonging to the Poaceae family including maize and *Sorghum* spp. [20,24,25]. The *B*. *fusca* larvae used stemmed from maize plants in Gilgil and Mahi-Mahiu (Rift Valley, Kenya). The egg to adult development time lasts about 60 days at 26 ± 1°C (Petit C., Pers. Obs.).

*Sesamia nonagrioides* Lefebvre is a polyphagous species found on more than 30 plant species belonging to the Poaceae, Cyperaceae and Typhaceae families [20–22]. The *S*. *nonagrioides* larvae used stemmed from *Typha domingensis* Pers. in Makindu (Eastern Kenya). The developmental time from egg to adult emergence is 52 days at 26 ± 1°C (Petit C., Pers. Obs.).

Colonies of each species were established on artificial diet [26] for more than 10 generations under laboratory conditions (26 ± 1°C and 50–60% RH) before being used in the experiments.

### Experimental procedures

**Fig 1:**
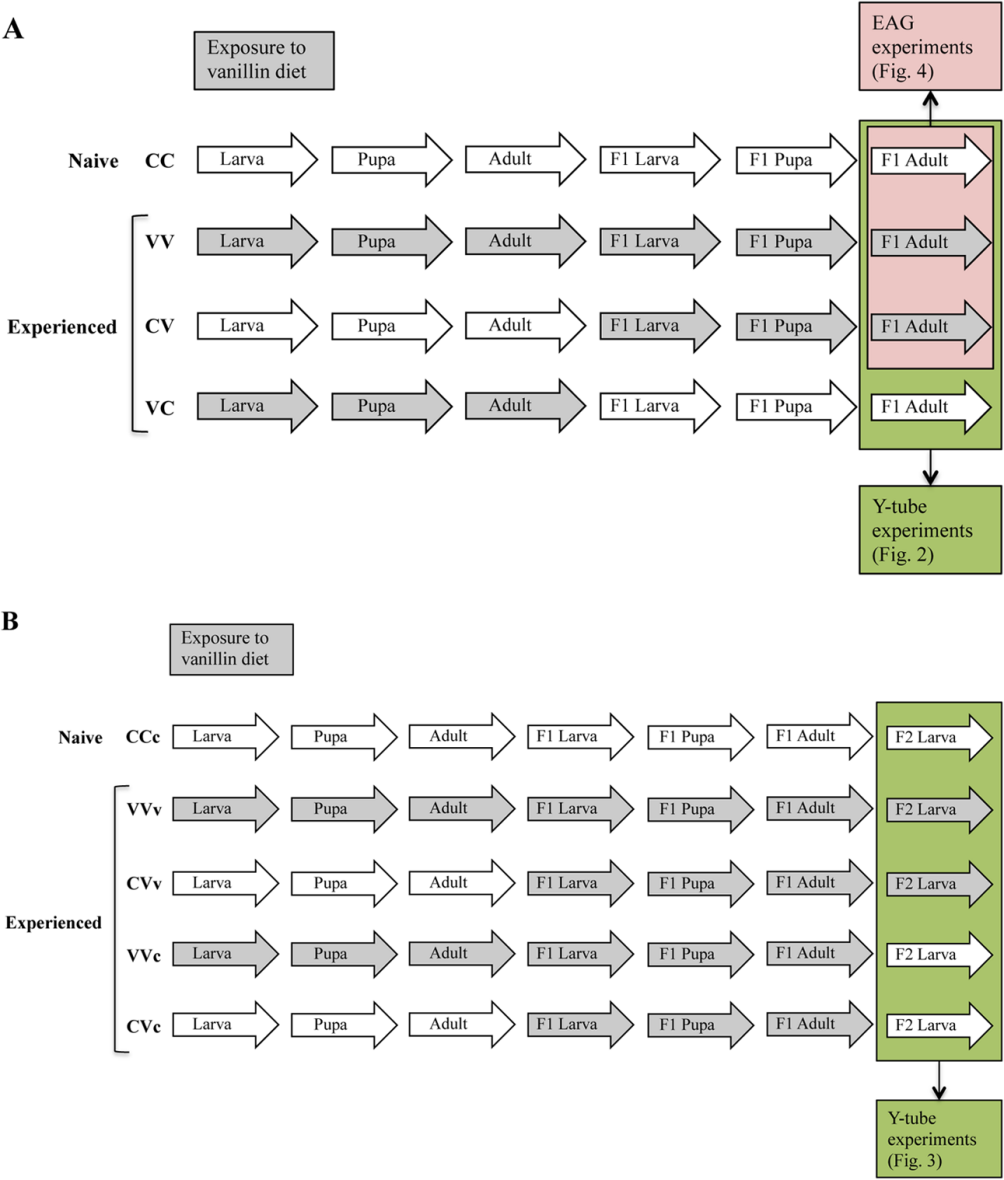
Description of the different conditioning procedures used for the experiments. (A) for mated female (B) for 3^rd^ instar larvae. Capital letters indicate for each cohort the type of diet on which they have been reared (C for the control diet; V for the same diet but enriched with vanillin). For 3^rd^ instar larvae, the last letter is in lower case because the insects were reared only up to the 3^rd^ instar on the control diet (c) or diet enriched with vanillin (v).

The insects were subjected to different conditioning procedures (Fig 1) using artificial diet enriched with vanillin (= vanillin diet), a compound not present in their natural hosts. Using a preliminary rearing experiment with larvae, the highest concentrations of vanillin not detrimental to larval survival and growth for each moth species were determined; they were 1g/l for *B*. *fusca* and *S*. *nonagrioides*, and 0.5 g/l for *B*. *nairobica*. *B*. *fusca* and *S*. *nonagrioides* were reared in plastic jars (16-cm-height, 9.5-cm-diameter) with ventilated lids. Each jar contained 200ml of artificial diet and was inoculated with 30 neonates. Because of a high mortality when reared in a jar, *B*. *nairobica* was reared in glass vials (7.4-cm-height, 2.5-cm-diameter) plugged with cotton wool. Each vial contained 12ml of artificial diet and was inoculated with two neonates. To prevent risk of odour contamination, the rearing on vanillin diet and on control diet was carried out in two separate rooms under a reversed photoperiod (12-h light/12-h dark), at 26 ± 1°C and 50–60% RH.

### Y-tube olfactometer

Olfactometric tests on mated females of *S*. *nonagrioides*, *B*. *fusca* and *B*. *nairobica* subjected to the conditioning procedures CC, VV, CV and VC (Fig 1; the first and the second capital letters indicate, respectively, the type of diet on which the parents and the mated females have been reared [C for the control diet; V for the vanillin diet]) were performed 2–4 h after onset of the scotophase, corresponding to the period of oviposition for each species (Calatayud P.-A., Pers. Obs.). The behaviour of 3^rd^ instar larvae from CCc, VVv, CVv, VVc and CVc (Fig 1; the last letter is in lower case because the insects were reared only up to the 3^rd^ instar on the control diet [c] or diet enriched with vanillin [v]) were analyzed after 5h of starvation. A Y-tube olfactometer, described by Ngi-Song et al. [27], which has been shown to be useful for demonstrating differences in attractiveness to odours in moths [28], was used (length of stem: 18 cm; length of each arm: 34 cm; diameter: 4 cm). The closed ends of each chamber were connected with tubing to each arm of the Y-tube. Clean air was drawn into the system over the sample through the arms of the olfactometer. The airflow was set at 15 cm s^-1^ per arm and measured by flow metres connected between the chambers and the activated charcoal. For 30 min prior to each test, air was left flowing through the olfactometer setup to reach equilibrium in the two chambers and the Y-tube stem. The Y-tube experiments were carried out at 25 ± 1°C and 50–60% RH. Mated females and 3^rd^ instar larvae were released individually into the stem of the Y- tube and allowed to choose between control diet (sample of 3cm^3^) and vanillin diet (sample of 3cm^3^) odours for a maximum of 10 min. A choice was recorded when the insect passed 5 cm from the intersection into one arm and remained motionless there for more than 20 s. Those that made no choice were also recorded. Every five insects, odour sources connections to the chambers were reversed to minimise any locational bias and the chambers were cleaned thoroughly with water. For each conditioning procedure, the percentage of insects that made a distinct choice was calculated.

### EAG recordings

Vanillin was diluted in dichloromethane to obtain four dilutions: 0.1, 1, 10 and 100μg/μl. Ten microliters of the dilution were applied to a piece of Whatman filter paper (10 x 9 mm) and inserted in a glass Pasteur pipette once the solvent had evaporated. The control stimulus was dichloromethane (DCM). EAG recordings were performed at room temperature on 2–4 days old females. The reference and recording glass capillary electrodes were filled with electrolytic solution. The reference electrode was inserted in the neck while the recording electrode covered the cut tip of the antennae. The signal was amplified (x20) with a Syntech UN-06 amplifier (The Netherlands). EAG analysis was carried out using Autospike software (Syntech, The Netherlands). Clean and humidified air was blown continuously over the antenna at a constant rate of 12ml s^-1^ and the antennae were stimulated for 0.5 s with 10 μl vanillin dilution. Odour stimuli were applied at 30s intervals to ensure full recovery of antennal receptors.

Each dilution was tested twice per antenna starting with the lowest concentration of 0.1 μg/μl and ending with the highest concentration of 100 μg/μl to prevent premature saturation of the odorant receptors.

To account for solvent (DCM) and other background effects, we subtracted the averaged EAG responses by DCM recorded before and after the four vanillin recordings, as described by Dickens [29].

### Statistical analysis

All statistical analyses were done in R (R Core Team 2013). For the Y-tube experiments, Fisher’s exact tests were applied to compare the number of insects preferring the vanillin or the control diet across treatments for each species. A Generalised Linear Model (GLM) was used to determine the global effect of the parent’s and self-experiences combined, in the Y-tube responses with binomial error distribution. For this, we assigned binary values to denominate the artificial diet used for each generation whereby 1 was used for *vanillin diet* and 0 for *control diet*.

For each species, repeated measures ANOVA was used to evaluate the effect of the conditioning procedure on the EAG dose-response curves from female antennae (factors studied were vanillin concentration and conditioning procedure with a female as a random effect). The data on EAG responses were first normalised as followed:

$$transformed\;EAG\;response=\sqrt{EAG\;response-\min(EAG\;response)}$$

## Results

### Female preference induction

**Fig 2:**
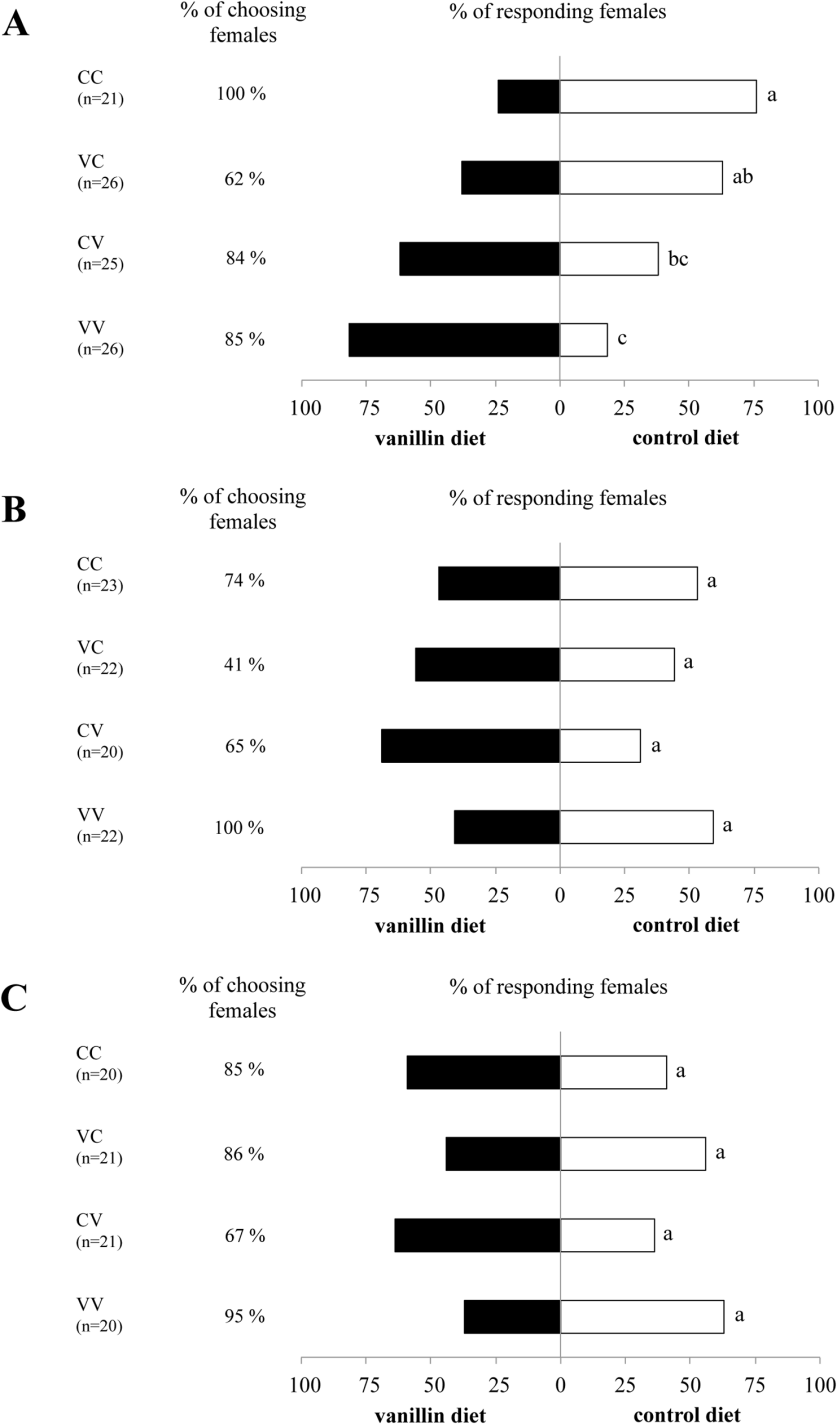
Response of mated female to odours of vanillin and control diets in a Y-tube olfactometer according to the different conditioning procedures CC, VC, CV and VV. (A) *S*. *nonagrioides* (B) *B*. *fusca* (C) *B*. *nairobica*. Between parentheses, the number n of mated females tested. The number of mated females making a choice was set to 100% to calculate the percentage of responding females. Bars with the same letter indicate no significant differences at 5% level between treatments according to Fisher’s exact test.

For the polyphagous species *S*. *nonagrioides*, when both the parents and themselves have been continuously exposed to the vanillin diet, the females oriented significantly towards odours of this diet, compared to the naïve females (i.e. without previous exposure to vanilline) (Fig 2A; females VV compared to females CC: P = 0.0002). The self-experience only was sufficient for the females to show a preference for the odours of the vanillin diet, (Fig 2A; females CV compared to females CC: P = 0.0278). However, when their parents and themselves had not been exposed continuously to the vanillin diet, the *S*. *nonagrioides* females behaved like the naïve ones (Fig 2A; females VC compared to females CC: P = 0.4753). The parent’s and self-experiences combined had a significant effect on the induction of preference (GLM for *S*. *nonagrioides*: z value = 3.543, P = 0.0004).

For the oligophagous and monophagous species, *B*. *fusca* and *B*. *nairobica*, there was no significant difference of response between the conditioning procedures (Fig 2B and 2C). For these species, the parent’s and self-experiences combined had no effect on the induction of preference (GLM for *B*. *fusca*: z value = -0.496, P = 0.620; for *B*. *nairobica*: z value = -1.323, P = 0.186).

### Larval preference induction

**Fig 3:**
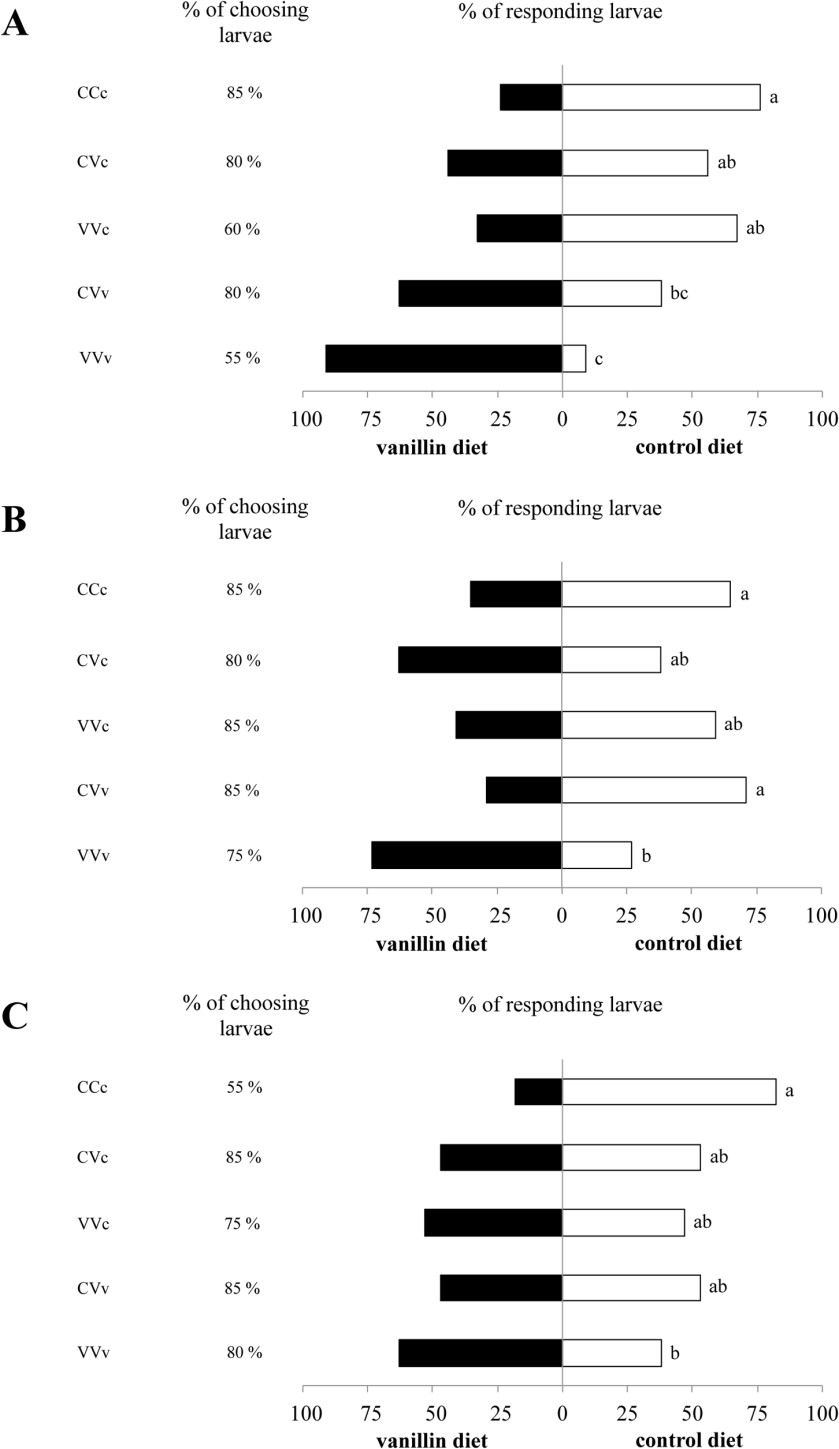
Response of 3^rd^ instar larvae to odours of vanillin and control diet in a Y-tube olfactometer according to the different conditioning procedures CCc, CVc, VVc, CVv and VVv. (A) *S*. *nonagrioides* (B) *B*. *fusca* (C) *B*. *nairobica*. Twenty 3^rd^ instar larvae were tested for each conditioning procedure. The number of larvae making a choice was set to 100% to calculate the percentage of responding larvae. Bars with the same letter indicate no significant differences at 5% level between treatments according to Fisher’s exact test.

For the three species, when the grandparents, parents and themselves had been continuously exposed to vanillin diet, the larvae showed a preference for the odours of this diet, compared to the naïve larvae (Fig 3A for *S*. *nonagrioides*, larvae VVv compared to larvae CCc: P = 0.0013; Fig 3B for *B*. *fusca*, larvae VVv compared to larvae CCc: P = 0.0416; Fig 3C for *B*. *nairobica*, larvae VVv compared to larvae CCc: P = 0.0473). However, only for *S*. *nonagrioides*, the parent’s and self-experiences were sufficient for the larvae to show a preference for the odours of the vanillin diet (Fig 3A; larvae CVv compared to larvae CCc: P = 0.0366).

For the polyphagous *S*. *nonagrioides*, the grandparent’s, parent’s and self-experiences combined had a significant effect on the induction of preference (GLM for *S*. *nonagrioides*: z value = 3.079, P = 0.0021), while they had no significant effect for the oligophagous and monophagous species, *B*. *fusca* and *B*. *nairobica* (GLM for *B*. *fusca*: z value = 1.225, P = 0.22; for *B*. *nairobica*: z value = 1.922, P = 0.0547).

### Female antennal sensitivity to vanillin

**Fig 4:**
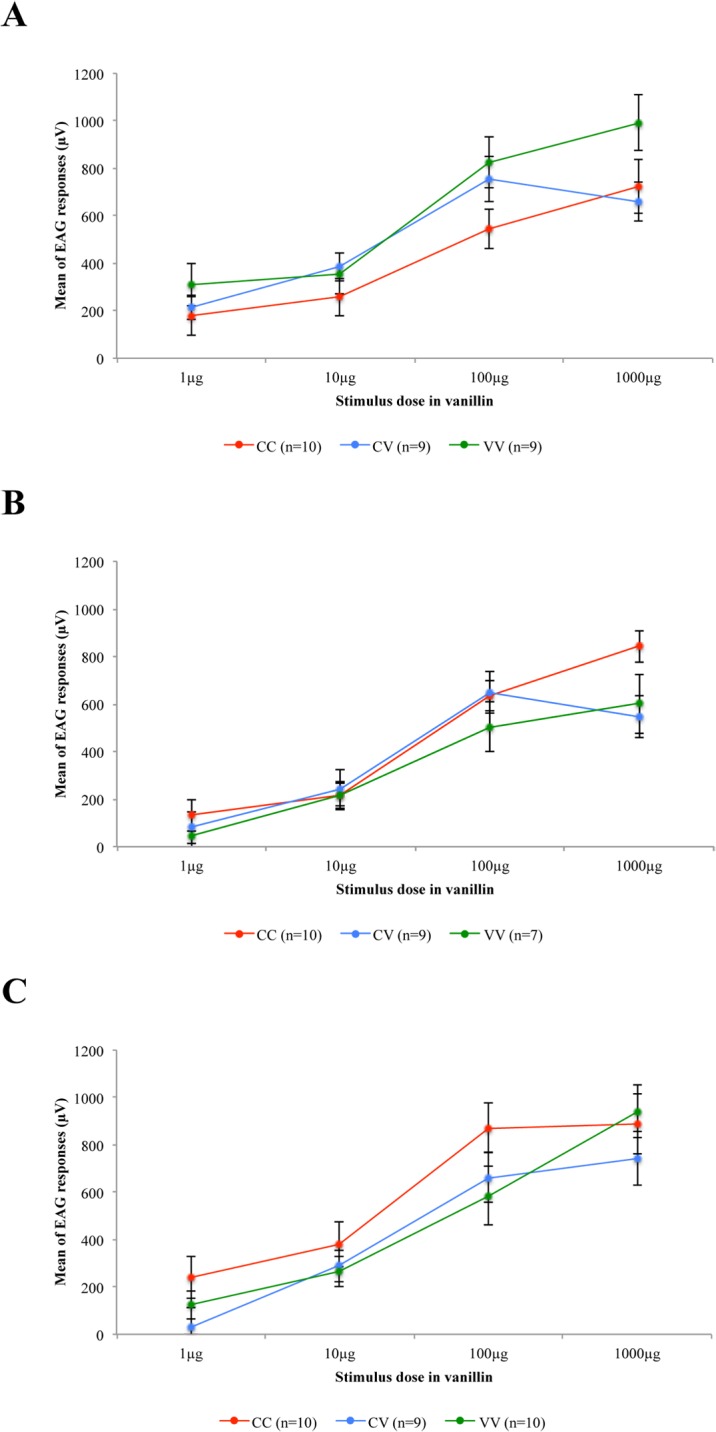
Dose-response curves of electrophysiological activity of the female antennae according to the three conditioning procedures CC, CV and VV. (A) *S*. *nonagrioides* (B) *B*. *fusca* (C) *B*. *nairobica*. Means (± SE) of EAG responses (maximum amplitudes of μV deflections) during the 0.5 s stimulation period with 1 to 1000μg of vanillin are given. For each species, repeated measures ANOVA was used to evaluate the effect of the conditioning procedure on the EAG dose-response curves from female antennae.

For each species, no significant difference was found in the EAG dose-response curves from female antennae between conditioning procedures within the dose range tested (Fig 4; repeated measures ANOVA for *S*. *nonagrioides*: F_2, 25_ = 1,81, P = 0.184; for *B*. *fusca*: F_2, 23_ = 1.066, P = 0.361 and for *B*. *nairobica*: F_2, 26_ = 1.115, P = 0.343).

## Discussion

This study illustrates how the olfactive dietary experience of neonate to adult insects with a new compound, vanillin, can induce adult and larval preference for vanillin-enriched diets. In adult females and 3^rd^ instar larvae of the polyphagous species *S*. *nonagrioides*, the preference for vanillin-enriched diets that appeared after one generation supports the Hopkins’ host selection principle, neo-Hopkins’ principle and chemical legacy.

The results indicate that *S*. *nonagrioides* displays a stronger phenotypic plasticity and thus accepts a new substrate more readily than oligophagous and monophagous species. The capacity to memorize new odour associated with food resources might explain why polyphagous insects are often important crop pests switching easily from the wild to the cultivated habitat [2]. This happened to African sugar cane borer *Eldana saccharina* Walker (Lepidoptera: Pyralidae), which switched from *Cyperus* sp. to sugar cane in South Africa and to maize in West Africa [30]. In an environment with large spatial and temporal heterogeneity, a behavioural phenotypic plasticity based upon experienced cues is advantageous over innate responses [5]. Thus, if environmental changes occur, *S*. *nonagrioides* has an advantage over *B*. *fusca* and *B*. *nairobica* because it can use experienced cues to adapt to such changes.

The results also suggest that the induction of preference for a new olfactory cue in *S*. *nonagrioides* is the result of the combination of parent’s experience and self-experience by the insects, indicating a transfer of information from adults to offspring linked with parent transmission. The influence of self-experience in insects on their preference induction for a particular host plant is well reported in the literature (see [11] for review); and the parent`s experience is known, for example, to affect the behaviour of the progeny through epigenetic effects [5,31] caused by a methylation of genes in gametes that pre-adapt the offspring to the environment [31,32].

After two generations of exposure to vanillin, the sensitivity of the antennae of the experienced females was the same as that of the naïve females, regardless of the moth species. Thus, the behavioural induction of preference for a new olfactory cue was not correlated with a higher sensitivity of the antennae.

This study showed that the polyphagous species *S*. *nonagrioides* exhibited a higher behavioural plasticity than the oligo- and monophagous species *B*. *fusca* and *B*. *nairobica*.

## References

[1] Schoonhoven LM, van Loon JJA, Dicke M. Insect-Plant Biology. 2005. 440 p.

[2] Bernays EA, Chapman RF. Host-plant selection by phytophagous insects. 1994.

[3] Berdegué M, Reitz SR, Trumble JT. Host plant selection and development in *Spodoptera exigua*: do mother and offspring know best? Entomol Exp Appl. 1998;89(1):57–64.

[4] Roessingh P, Xu S, Menken SBJ. Olfactory receptors on the maxillary palps of small ermine moth larvae: evolutionary history of benzaldehyde sensitivity. J Comp Physiol A. 2007;193:635–47. 10.1007/s00359-007-0218-x PMC1915583 17372741

[5] Anderson P, Anton S. Experience-based modulation of behavioural responses to plant volatiles and other sensory cues in insect herbivores. Plant, Cell Environ. 2014;37:1826–35. 24689897 10.1111/pce.12342

[6] Chakraborty TS, Goswami SP, Siddiqi O. Sensory correlates of imaginal conditioning in *Drosophila melanogaster* . J Neurogenet. 2009 1;23(1–2):210–9. 10.1080/01677060802491559 19058083

[7] Hopkins AD. Economic investigations of the scolytid bark and timber beetles of North America. US Dep Agric Progr Work. 1916;353.

[8] Akhtar Y, Isman MB. Larval exposure to oviposition deterrents alters subsequent oviposition behavior in generalist, *Trichoplusia ni* and specialist, *Plutella xylostella* moths. J Chem Ecol. 2003 8;29(8):1853–70. 12956511 10.1023/a:1024802328458

[9] Hora KH, Roessingh P, Menken SBJ. Inheritance and plasticity of adult host acceptance in *Yponomeuta* species: implications for host shifts in specialist herbivores. Entomol Exp Appl. 2005 4;115(1):271–81.

[10] Anderson P, Sadek MM, Larsson M, Hansson BS, Thöming G. Larval host plant experience modulates both mate finding and oviposition choice in a moth. Anim Behav. Elsevier Ltd; 2013 6;85(6):1169–75.

[11] Barron AB. The life and death of Hopkins’ host-selection principle. J Insect Behav. 2001;14(6):725–37.

[12] Corbet SA. Insect chemosensory responses: a chemical legacy hypothesis. Ecol Entomol. 1985;10(2):143–53.

[13] Jaenike J. Induction of host preference in *Drosophila melanogaster* . Oecologia. 1983 6;58(3):320–5. 28310329 10.1007/BF00385230

[14] Traynier RMM. Associative learning in the ovipositional behaviour of the cabbage butterfly, *Pieris rapae* . Physiol Entomol. 1984 12;9(4):465–72.

[15] Cunningham JP, Jallow MFA, Wright DJ, Zalucki MP. Learning in host selection in *Helicoverpa armigera* (Hübner) (Lepidoptera: Noctuidae). Anim Behav. 1998;55:227–34. 9480690 10.1006/anbe.1997.0600

[16] Zhang P-J, Liu S-S, Wang H, Zalucki MP. The influence of early adult experience and larval food restriction on responses toward non host plants in moths. J Chem Ecol. 2007 8;33(8):1528–41. 17593465 10.1007/s10886-007-9325-y

[17] Chow JK, Akhtar Y, Isman MB. The effects of larval experience with a complex plant latex on subsequent feeding and oviposition by the cabbage looper moth: *Trichoplusia ni* (Lepidoptera: Noctuidae). Chemoecology. 2005 5 30;15(3):129–33.

[18] Bernays EA. Neural limitations in phytophagous insects: implications for diet breadth and evolution of host affiliation. Annu Rev Entomol. 2001;46:703–27. 11112184 10.1146/annurev.ento.46.1.703

[19] Felix A-E, Calatayud P-A, Le Ru B, Capdevielle-Dulac C, Ong’amo G, Silvain J-F, et al To be or not to be a species: use of reproductive isolation experiments and genetic analysis to clarify the taxonomic status of two *Busseola* (Lepidoptera: Noctuidae) species in Kenya. Ann la Société Entomol Fr. 2013;49(3):345–54.

[20] Le Ru BP, Ong’amo G, Moyal P, Muchugu E, Ngala L, Musyoka B, et al Geographic distribution and host plant ranges of East African noctuid stem borers. Ann la Société Entomol Fr. 2006;42(3–4):353–61.

[21] Le Ru BP, Ong’amo GO, Moyal P, Ngala L, Musyoka B, Abdullah Z, et al Diversity of lepidopteran stem borers on monocotyledonous plants in eastern Africa and the islands of Madagascar and Zanzibar revisited. Bull Entomol Res. 2006;96:555–63. 17201973 10.1017/ber2006457

[22] Ong’amo GO, Le Ru BP, Dupas S, Moyal P, Calatayud P-A, Silvain J-F. Distribution, pest status and agro-climatic preferences of lepidopteran stem borers of maize in Kenya. Ann la Société Entomol Fr. 2006;42(2):171–7.

[23] Ong’amo GO. Diversity, ecology and population dynamics of lepidopteran stem borers in Kenya. 2009.

[24] Ndemah R, Schulthess F, Le Rü B, Bame I. Lepidopteran cereal stemborers and associated natural enemies on maize and wild grass hosts in Cameroon. J Appl Entomol. 2007;131(9–10):658–68.

[25] Moolman J, Van den Berg J, Conlong D, Cugala D, Siebert S, Le Ru B. Species diversity and distribution of lepidopteran stem borers in South Africa and Mozambique. J Appl Entomol. 2014;138:52–66.

[26] Onyango FO, Ochieng’-Odero JPR. Continuous rearing of the maize stem borer *Busseola fusca* on an artificial diet. Entomol Exp Appl. 1994;73:139–44.

[27] Ngi-Song AJ, Overholt WA, Njagi PGN, Dicke M, Ayertey JN, Lwande W. Volatile infochemicals used in host an host habitat location by *Cotesia flavipes* Cameron and *Cotesia sesamiae* (Cameron) (Hymenoptera: Braconidae), larval parasitoids of stemborers on graminae. J Chem Ecol. 1996;22(2):307–23. 10.1007/BF02055101 24227412

[28] Calatayud P-A, Ahuya P, Le Ru B. Importance of the experimental setup in research on attractiveness of odours in moths: an example with *Busseola fusca* . Entomol Exp Appl. 2014 7 18;152(1):72–6.

[29] Dickens JC. Olfaction in the boll weevil, *Anthonomus grandis* Boh. (Coleoptera: Curculionidae): electroantennogram studies. J Chem Ecol. 1984;10(12):1759–85. 10.1007/BF00987360 24318432

[30] Atkinson PR. On the biology, distribution and natural host-plants of *Eldana saccharina* Walker (Lepidoptera: Pyralidae). J Entomol Soc South Africa. 1980;43(2):171–94.

[31] Ledón-Rettig CC, Richards CL, Martin LB. Epigenetics for behavioral ecologists. Behav Ecol. 2013;24(2):311–24.

[32] Youngson NA, Whitelaw E. Transgenerational epigenetic effects. Annu Rev Genomics Hum Genet. 2008;9:233–57. 10.1146/annurev.genom.9.081307.164445 18767965

